# Integrative Analysis of Transcriptome-Wide Association Study and Gene-Based Association Analysis Identifies In Silico Candidate Genes Associated with Juvenile Idiopathic Arthritis

**DOI:** 10.3390/ijms232113555

**Published:** 2022-11-04

**Authors:** Shuai Liu, Weiming Gong, Lu Liu, Ran Yan, Shukang Wang, Zhongshang Yuan

**Affiliations:** 1 Department of Biostatistics, School of Public Health, Cheeloo College of Medicine, Shandong University, Jinan 250012, China; 2 Institute for Medical Dataology, Cheeloo College of Medicine, Shandong University, Jinan 250012, China

**Keywords:** juvenile idiopathic arthritis, transcriptome-wide association study, gene-based association analysis, enrichment analysis

## Abstract

Genome-wide association study (GWAS) of Juvenile idiopathic arthritis (JIA) suffers from low power due to limited sample size and the interpretation challenge due to most signals located in non-coding regions. Gene-level analysis could alleviate these issues. Using GWAS summary statistics, we performed two typical gene-level analysis of JIA, transcriptome-wide association studies (TWAS) using FUnctional Summary-based ImputatiON (FUSION) and gene-based analysis using eQTL Multi-marker Analysis of GenoMic Annotation (eMAGMA), followed by comprehensive enrichment analysis. Among 33 overlapped significant genes from these two methods, 11 were previously reported, including TYK2 (*P*_FUSION_ = 5.12 × 10^−6^, *P*_eMAGMA_ = 1.94 × 10^−7^ for whole blood), IL-6R (*P*_FUSION_ = 8.63 × 10^−7^, *P*_eMAGMA_ = 2.74 × 10^−6^ for cells EBV-transformed lymphocytes), and Fas (*P*_FUSION_ = 5.21 × 10^−5^, *P*_eMAGMA_ = 1.08 × 10^−6^ for muscle skeletal). Some newly plausible JIA-associated genes are also reported, including IL-27 (*P*_FUSION_ = 2.10 × 10^−7^, *P*_eMAGMA_ = 3.93 × 10^−8^ for Liver), LAT (*P*_FUSION_ = 1.53 × 10^−4^, *P*_eMAGMA_ = 4.62 × 10^−7^ for Artery Aorta), and MAGI3 (*P*_FUSION_ = 1.30 × 10^−5^, *P*_eMAGMA_ = 1.73 × 10^−7^ for Muscle Skeletal). Enrichment analysis further highlighted 4 Kyoto Encyclopedia of Genes and Genomes (KEGG) pathways and 10 Gene Ontology (GO) terms. Our findings can benefit the understanding of genetic determinants and potential therapeutic targets for JIA.

## 1. Introduction

Juvenile idiopathic arthritis (JIA) is one of the most common rheumatic diseases characterized by arthritis in childhood [1]. It can cause damage to multiple organs such as arthrosis, heart, lung, skin, and eyes, and is an important cause of disability in children under the age of 16 [2]. Previous studies have shown that the concordance rate of JIA in monozygotic twins and the relative risk of the disease in the siblings of JIA patients are higher [3,4], highlighting the important role of genetic factors in the development of JIA [5,6]. Therefore, it is of great significance to probe the complex genetic association of JIA to better understand the genetic mechanisms and investigate potential intervention targets.

Genome-wide association studies (GWAS) have successfully identified 22 risk loci associated with JIA [7,8,9], however, these GWASs suffer from either small sample size or low case proportions, which may be presumably due to the difficulty in accurate JIA diagnosis and the lack of pathognomonic features [10]. So far, the JIA GWAS with relatively larger sample size and the largest case proportions only includes 3305 JIA cases and 9196 controls [9]. In addition, most genetic variants identified from GWAS of JIA are located in non-coding regions [11], leading to the difficulty in explaining the association signals. On the other hand, statistically, GWAS often provide JIA-associated single nucleotide polymorphisms (SNPs), the effects of which are too weak to be detected. Gene-level analysis can not only aggregate many SNPs with small effects to improve the power, but provide good biological interpretations, which is more straightforward to be translated into clinical practice.

With the increase of publicly available GWAS summary data and the well-developed efficient tools, it is feasible to conduct the gene-level analysis for JIA [12]. There are two typical gene-level association analysis methods with different model assumptions, one is transcriptome-wide association studies (TWAS), which has shown great promise in interpreting the GWAS signals and is powerful in detecting the association between the gene expression level and the complex disease [13,14]. Recently, one TWAS analysis of JIA has been conducted, however, it only involves two tissues with lower JIA case proportions [15], which may lead to the power loss and the insufficiency in capturing the tissue information related with JIA. The other is multi-marker analysis, which can assign SNPs to genes based on physical proximity and further conduct gene-based association analysis. Both methods, though have different statistical principles, can produce the gene-level *p* values. We would like to emphasize that using different gene-level methods with different model statistical principles to obtain the common genes can avoid the risk of false discoveries from using single method. Actually, the results from different analysis could complement to each other [16,17].

In the present study, using the GWAS summary data of JIA with relatively larger sample size and case proportions (3305 cases and 9196 controls), we performed TWAS analysis and the gene-based association analysis to identify the tissue-specific JIA-associated genes. We used the false discovery rate (FDR) correction on each tissue to declare the significant genes. Finally, we overlapped the genes significantly detected from these two gene-level methods, and performed the enrichment analysis for these overlapped genes on the Metascape website to identify the significant Gene Ontology (GO) terms as well as the Kyoto Encyclopedia of Genes and Genomes (KEGG) pathways.

## 2. Methods

### 2.1. Study Design, Data Source, and Quality Control

**Figure 1:**
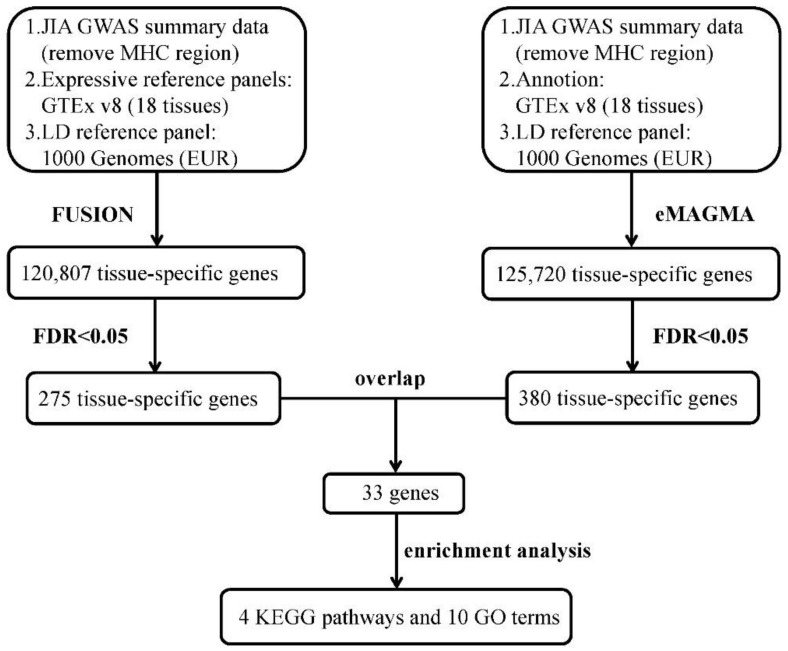
The flowchart of integrative analysis of FUSION and eMAGMA. ABBR: JIA, Juvenile idiopathic arthritis; GWAS, genome-wide association study; MHC, major histocompatibility complex; GTEx, Genotype-Tissue Expression Project; LD, linkage disequilibrium; EUR, European; FUSION, functional summary-based imputation; eQTL, expression quantitative trait loci; eMAGMA, eQTL Multi-marker Analysis of GenoMic Annotation; FDR, false discovery rate; KEGG, Kyoto Encyclopedia of Genes and Genomes; GO, Gene Ontology.

The analysis flowchart of this study is shown in Figure 1. We used the GWAS of JIA from a large-scale meta-analysis [9]. We obtained the largest GWAS summary statistics of JIA from the NHGRI-EBI GWAS Catalog (Study Accession Code GCST90010715), where the JIA cases were diagnosed according to The International League Against Rheumatism (ILAR) criteria. The data were restricted to European ancestry with stringent quality control as described previously [9]. Briefly, the GWAS of JIA initially recruited 4520 UK JIA patients and 9965 healthy individuals. Individuals with call rate less than 0.98 and discrepancy between genetically predicted sex and database record were removed. In addition, SNPs that were non-autosomal, had a call rate <0.98 or a minor allele frequency (MAF) <0.01 were further excluded. About 12,501 individuals (3305 cases and 9196 healthy controls) were finally remained. For the summary data, we further excluded the major histocompatibility complex (MHC) region (chromosome 6: 25–35 Mb) due to its complex structure, restricted to biallelic SNPs and removed SNPs with duplicated or missing rs ID for subsequent analyses. Totally 7,415,262 SNPs are finally included. Bearing in mind that using different methods with different model assumptions to obtain the overlapped genes can avoid the risk of false discoveries from using single method, we here applied two gene-level approaches with distinct principles, TWAS analysis and gene-based association analysis, as parallel analyses to obtain the common JIA-associated genes for enrichment analysis.

### 2.2. TWAS Analysis

TWAS aim to integrate GWAS and expression quantitative trait loci (eQTL) studies to identify tissue-specific gene-trait associations [18], which has shown great promise both in interpreting GWAS findings and in elucidating the underlying disease mechanisms. We here adopted FUnctional Summary-based ImputatiON (FUSION) method (http://gusevlab.org/projects/fusion/, accessed on 8 April 2022) to conducted TWAS analysis of JIA. FUSION is the most commonly used TWAS method and has shown great promise in large-scale integrative omics data analysis [14]. Once inputting the GWAS summary data and expression weight, FUSION will impute the gene expressions in GWAS, and then perform an association analysis between the predicted gene expression and JIA. We selected Genotype-Tissue Expression Project (GTEx) v8 with pre-computed gene expression weights from totally 49 tissues. Using all the tissues may introduce the nuisance information and increase the computation burden; previous studies recommend using an expression panel from only plausible disease-related tissues in TWAS [13]. Here, we determine the analyzed tissue based on not only previous studies [2,19], but also the clinical symptoms and involved organs of JIA, such as hepatomegaly, splenomegaly, anemia, disseminated intravascular coagulation, arthritis, rash, mesenteric lymphadenopathy, pericarditis, and pneumonia. We finally selected 18 tissues for analysis including artery aorta, artery coronary, artery tibial, cells EBV-transformed lymphocytes, colon sigmoid, colon transverse, heart atrial appendage, heart left ventricle, liver, lung, muscle skeletal, nerve tibial, skin not sun exposed suprapubic, skin sun exposed lower leg, small intestine terminal ileum, spleen, stomach, whole blood. We used the 1000 Genomes European panel as a linkage disequilibrium (LD) reference data, and obtained tissue-specific *p* value for each gene across different tissues. We finally performed FDR correction on each tissue, and genes with FDR less than 0.05 are declared to be significant.

### 2.3. Gene-Based Association Analysis

We used eQTL Multi-marker Analysis of GenoMic Annotation (eMAGMA) method (https://github.com/eskederks/eMAGMA-tutorial, accessed on 28 March 2022) to conduct gene-based association analysis for JIA. eMAGMA follows the same statistical framework as MAGMA, which is based on a multiple linear principal component regression model and can provide better statistical performance in gene-based association analysis [16]. Besides this, eMAGMA can further utilize tissue-specific cis-eQTL information to assign SNPs to putative genes, providing more biologically meaningful and interpretable results [17]. Gene-based analysis using eMAGMA typically involves two stages, annotation and gene analysis [16]. We, in the annotation stage, used the same tissues as that in above TWAS analysis and directly used the GTEx v8-based annotation files provided on eMAGMA′s website. In the gene analysis stage, we used the 1000 Genomes European panel as the reference panel and tested the association between the annotated genes and JIA. We further performed FDR correction on each tissue, and selected significant genes with FDR less than 0.05.

### 2.4. Gene Set Enrichment Analysis

We conducted gene set enrichment analysis for the overlapped genes that were significantly identified by both TWAS and eMAGMA analysis. Specifically, these overlapped significant genes were subjected to GO term and KEGG pathway enrichment analysis on the Metascape website (https://metascape.org/gp/index.html#/main/step1, accessed on 26 April 2022) to better understand the biological mechanisms. Metascape essentially utilizes the hypergeometric test and Benjamini-Hochberg *p* value correction algorithm to identify all ontology terms. A large number of terms would make the results redundant and complicate the interpretation, Kappa consistency test was thus performed and terms with Kappa > 0.3 were grouped into a cluster, and the most statistically significant term in the cluster was selected to represent the cluster [20]. The parameters of Min Overlap, *p* Value Cutoff, and Min Enrichment are set to be the default values, respectively. In addition, we also made a protein–protein interaction (PPI) network for the overlapped significant genes on the STRING website (https://cn.string-db.org/, accessed on 15 September 2022).

## 3. Results

### 3.1. TWAS Analysis

We analyzed all genes involved in the 18 tissues, among which 275 tissue-specific genes were significantly detected by FUSION with FDR less than 0.05. Note that LAT is significantly detected at the border line (*p* = 1.53 × 10^−4^ and FDR = 5.40 × 10^−2^ for Artery Aorta). These TWAS significant genes included some established JIA-associated genes that have been reported previously, such as CCDC101 (*p* = 5.82 × 10^−8^ and FDR = 1.64 × 10^−4^ for Muscle Skeletal), CLN3 (*p* = 5.82 × 10^−8^ and FDR= 2.53 × 10^−4^ for Whole Blood), ERAP2 (*p* = 5.49 × 10^−6^ and FDR = 2.16 × 10^−3^ for Cells EBV-transformed lymphocytes), LNPEP (*p* = 3.53 × 10^−6^ and FDR = 2.36 × 10^−3^ for Artery Tibial). The consistent results with previous GWAS partly indicates the correctness of the FUSION analysis. All significant genes in FUSION analysis were displayed in Supplementary Table S1.

### 3.2. Gene-Based Association Analysis Results

Similarly, we analyzed all genes involved in the 18 tissues, among which 380 tissue-specific genes were significantly detected by eMAGMA with FDR less than 0.05. These included some well-known JIA-associated genes, such as SGF29 (*p* = 2.22 × 10^−8^ and FDR = 3.38 × 10^−5^ for Whole Blood), ANKRD55 (*p* = 3.09 × 10^−8^ and FDR = 1.08 × 10^−4^ for Spleen), ATP8B2 (*p* = 7.53 × 10^−7^ and FDR = 6.59 × 10^−4^ for Stomach), PTPN2 (*p* = 3.96 × 10^−6^ and FDR = 2.75 × 10^−3^ for Spleen). Again, all these genes are included in the 22 risk loci identified by previous GWAS illustrate the eMAGMA results more reliable. All significant genes from eMAGMA analysis were summarized in Supplementary Table S2.

### 3.3. Gene Set Enrichment Analysis

We intersected the significant genes from both TWAS analysis and eMAGMA gene-based association analysis according to different tissues, and found a total of 132 tissue-specific genes. A total of 33 unique genes were further identified after removing the duplicated ones, among which 11 genes have been reported in previous studies [7,8,9], such as TYK2 (*P*_FUSION_ = 5.12 × 10^−6^*, P_eMAGMA_ =* 1.94 × 10^−7^ for Whole Blood), IL(Interleukin)-6R (*P*_FUSION_ = 8.63 × 10^−7^*, P_eMAGMA_ =* 2.74 × 10^−6^ for Cells EBV-transformed lymphocytes), and Fas (*P*_FUSION_ = 5.21 × 10^−5^*, P_eMAGMA_ =* 1.08 × 10^−6^ for Muscle Skeletal). The remaining newly discovered 22 genes are APOBR, ATXN2L, GSDMB, IKZF3, IL27, KIAA1109, LAT, LMAN2L, MAGI3, NFATC2IP, NUPR1, ORMDL3, PHTF1, PSMB7, RGS14, SBK1, SH2B1, STEAP1B, TNFSF15, TUFM, UXS1, ZNF197. Among them, IL-27 (*P*_FUSION_ = 2.10 × 10^−7^*, P_eMAGMA_ =* 3.93 × 10^−8^ for Liver), LAT (*P*_FUSION_ = 1.53 × 10^−4^*, P_eMAGMA_ =* 4.62 × 10^−7^ for Artery Aorta) and MAGI3 (*P*_FUSION_ = 1.30 × 10^−5^*, P_eMAGMA_ =* 1.73 × 10^−7^ for Muscle Skeletal) are the novel genes that are more likely to be associated with JIA. Detailed information for 33 genes were summarized in Table 1.

**Figure 2:**
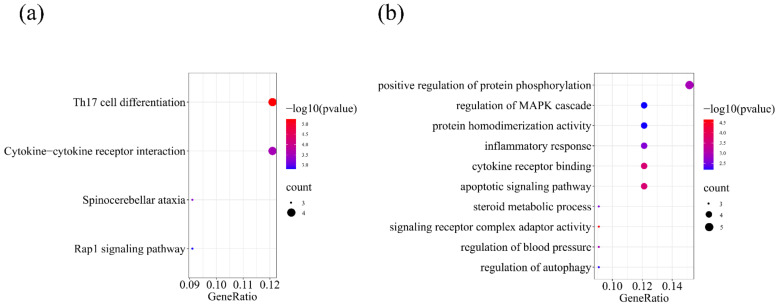
KEGG and GO enrichment analysis of 33 overlapped genes by Metascape. (**a**) Bubble chart for KEGG enrichment analysis. (**b**) Bubble chart for GO enrichment analysis.

**Figure 3:**
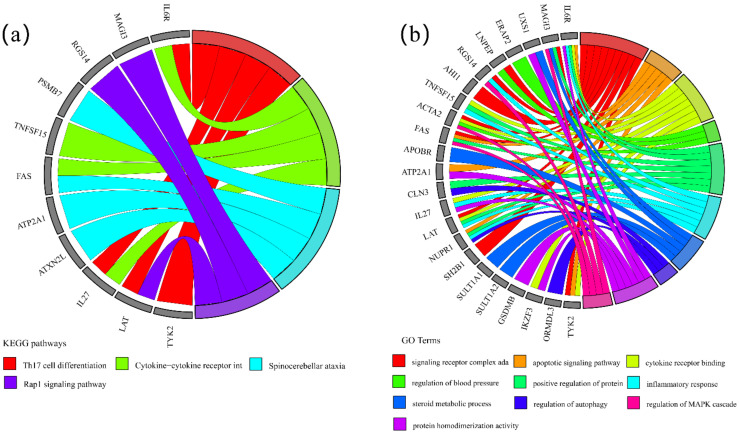
Chord graphs for four significant KEGG pathways and ten significant GO terms. (**a**) Chord graph of four significant KEGG pathways. (**b**) Chord graph of ten significant GO terms. For each panel, the right semicircle represented significant pathways or terms, and the left semicircle represented the genes enriched in these pathways or terms.

**Figure 4:**
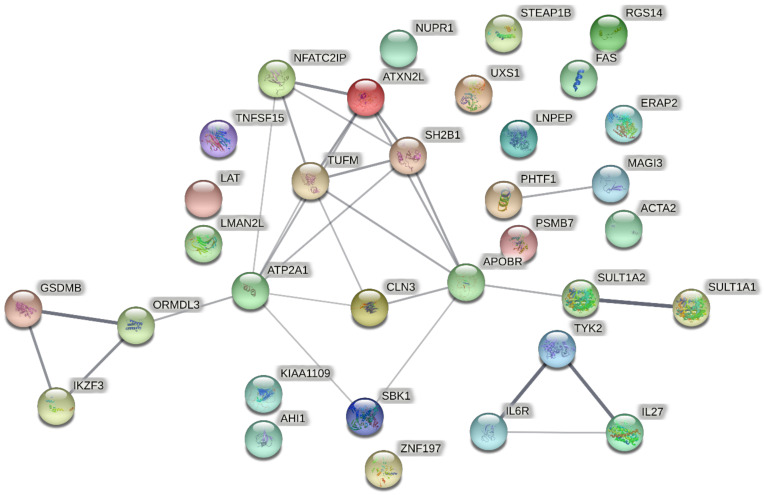
Protein–protein interaction (PPI) network for 33 overlapped genes by STRING. Each circle represents a protein, a line between proteins indicates PPI, line thickness indicates the strength of data support.

We further performed KEGG and GO enrichment analysis on the overlapped 33 significant genes on the Metascape website, respectively. For KEGG enrichment analysis, we totally found four significant KEGG pathways (Figure 2), including Th17 cell differentiation (*p =* 5.83 × 10^−6^), cytokine–cytokine receptor interaction (*p =* 2.92 × 10^−4^), spinocerebellar ataxia (*p =* 5.11 × 10^−4^), and Rap1 signaling pathway (*p =* 1.55 × 10^−3^). For GO enrichment analysis, we totally found ten significant GO terms (Figure 2), including signaling receptor complex adaptor activity (*p =* 2.25 × 10^−5^), apoptotic signaling pathway (*p =* 2.09 × 10^−4^), cytokine receptor binding (*p = 2.14* × 10^−4^), regulation of blood pressure (*p =* 1.16 × 10^−3^), positive regulation of protein phosphorylation (*p =* 1.18 × 10^−3^), inflammatory response (*p = 2.44* × 10^−3^), steroid metabolic process (*p =* 2.66 × 10^−3^), regulation of autophagy (*p =* 5.96 × 10^−3^), protein homodimerization activity (*p =* 6.40 × 10^−3^), and regulation of MAPK cascade (*p =* 6.47 × 10^−3^). In addition, chord graphs (Figure 3) were also depicted to show the most significant enrichment pathways of KEGG and GO and visualize the targeting relationship between significant genes and significant pathways, so as to visualize which pathways every gene is enriched to, and which genes are enriched in each pathway. PPI network (Figure 4) shows the interaction between proteins.

## 4. Discussion

In the present study, we performed a comprehensive large-scale gene-level analysis using co-complementary methods and successfully detected 33 common genes, including 11 previously reported genes such as TYK2, IL-6R, and Fas [7,8,9], and 22 novel potential genes such as IL-27, LAT, and MAGI3. Enrichment analysis suggested important role of pathways involving Th17 cell differentiation and Rap1 signaling pathway, followed by PPI network illustrating the protein–protein interactions. All these findings provide novel insights into the potential molecular mechanisms underlying the development of JIA.

TYK2, IL-6R, and Fas appear more frequently in the significant KEGG pathways and Go terms, and the expression products of these three genes have been shown to play important roles in the inflammatory and immune responses of JIA [21,22,23]. Tyrosine kinase 2 encoded by gene TYK2 is a part of janus kinase (JAK), which mediates the activation of signal transducers and activators of transcription (STAT) proteins. That is, TYK2 may play a role in autoimmunity and inflammation through abnormal expression in JAK-STAT pathway, thus leading to JIA [21,24,25]. IL-6R encodes part of the interleukin 6 receptor, as a pro-inflammatory cytokine, IL-6 is significantly elevated in the serum of JIA patients. Inhibition of IL-6R expression reduces IL-6 and IL-6 receptor binding, thereby reducing inflammation and immune responses in JIA patients [22,26]. Fas can induce T-cell apoptosis by binding to the Fas Ligand (FasL), so decreased gene Fas expression may lead to the accumulation of activated T-cells and cause autoimmune diseases [27].

IL-27 is involved in encoding the synthesis of IL-27, a cytokine that plays a role in innate immunity and whose primary function is to promote pro-inflammatory Th1 differentiation and inhibit anti-inflammatory Th2 responses [28,29,30,31,32]. IL-27 promotes Th1 cell differentiation, which in turn produces a large amount of the proinflammatory cytokine interferon-γ (IFN-γ) to play a pathogenic role in JIA [32,33]. The promotion of Th1 differentiation and the inhibition of Th2 differentiation by IL-27 is also dependent on the action of STAT1 [28,31,32,33], that is, the role of IL-27 is also involved in the JAK-STAT signaling pathway. Therefore, if the expression of gene IL-27 is inhibited, the inflammatory response of JIA patients can be correspondingly reduced.

LAT encodes a protein called T-cell activation adaptor [34]. T-cell receptor (TCR) signaling is an important process in T-cell development and its activation in the periphery [35]. LAT is a key signaling hub connecting TCRs to trigger downstream T-cell responses. If LAT gene expression is reduced, peripheral T-cell development and numbers are inhibited. Decreased numbers of T cells are prone to lead to immunodeficiency and autoimmune diseases [36], and patients with JIA are likely to have decreased autoimmune function due to lack of LAT.

MAGI3 encodes Membrane-associated guanylate kinase, WW and PDZ domain-containing protein 3. Abnormal expression of MAGI3 may affect Notch signaling and thus affect bone and joint development in children [37]. In addition, MAGI3 is also a risk gene for rheumatoid arthritis (RA), Graves′ disease, and other autoimmune diseases, indicating it can also cause JIA by affecting the human immune system [38,39].

Enrichment analysis suggested important role of pathways involving Th17 cell differentiation and Rap1 signaling pathway. The Th17 cell differentiation pathway is involved in inflammation and bone destruction, IL-27, IL-6R, LAT, TYK2 are all on this pathway. Th17 cells are actively differentiated and mainly secrete IL-17, which not only promotes the production of inflammatory cytokines in the JAK-STAT signaling pathway, but also catalyzes the maturation of osteoclasts, leading to osteopenia and joint damage [40,41]. The activation of inflammatory cytokines and osteopenia together lead to arthritis, and so inhibiting the differentiation of Th17 cells may inhibit and treat JIA to a certain extent [42,43,44].

The Rap1 signaling pathway plays a central role in the functional outcome of T-cell stimulation [45]. Rap1 is a T-cell receptor proximal signaling protein, and its abnormal expression may lead to abnormal T cells [46]. T cells activate macrophages and synovial stromal cells pleiotropically through cell-to-cell contact and interleukin production, leading to synovitis and joint destruction in RA [47,48]. The pathogenic behavior of the above T cells is caused by Rap1 inactivation, and sustained Rap1 signaling in T cells can effectively reduce the incidence and severity of arthritis [49,50]. Therefore, activation and enhancement of Rap1 signaling also contribute to the prevention and treatment of JIA.

Our study is not without limitations. First, we only focused on European ancestry due to the current large-scale GWASs of JIA was only available for European population. The findings cannot be directly generalized to other ethnic population. Second, the results from data analysis are often less reliable than that from serious and high-cost experiments, which is likely to be the gold standard in biomedicine studies. However, the data analysis is still valuable. For example, it is often hard to pre-specify the experimental target under a hypothesis-free approach, data analysis can help to narrow down the candidate experimental target list and provide the evidence of target priority. In addition, the current analysis pipeline can be easily extended, an alternative way is to search for restriction endonuclease (RE) sites in the non-coding regions and gain insights through RE digestion patterns [51,52]. Third, our findings are obtained from a joint analysis of all subtypes of JIA, and there is no guarantee that the conclusions would be valid for any subtype. Finally, the results must necessarily be confirmed by experiments in the laboratory, given that all the analysis are essentially in silico.

## 5. Conclusions

In summary, we performed gene-level analysis as well as enrichment analysis on the largest GWAS summary data of JIA. We identified novel JIA-associated genes including IL-27, LAT, and MAGI3, and highlight the important role of Th17 cell differentiation, Rap1 signaling pathway in the development of JIA. Our results can provide new insights into the pathogenic mechanisms as well as potential therapeutic targets of JIA; however, further studies are still required to validate these findings.

## Figures and Tables

**Table 1:** Overlapped gene identified by FUSION and eMAGMA.

Gene Symbol	Chromosome	Gene Start (bp)	Gene End (bp)	*P*_FUSION_	FDR_FUSION_	*P*_eMAGMA_	FDR_eMAGMA_
IL6R	1	154,377,669	154,441,926	8.63 × 10^−7^	7.80 × 10^−4^	2.74 × 10^−6^	1.56 × 10^−3^
**MAGI3**	1	113,933,371	114,228,545	1.30 × 10^−5^	7.35 × 10^−3^	1.73 × 10^−7^	2.62 × 10^−4^
**PHTF1**	1	114,239,453	114,302,111	6.55 × 10^−7^	1.21 × 10^−3^	2.93 × 10^−7^	5.60 × 10^−4^
**LMAN2L**	2	97,371,666	97,405,801	4.16 × 10^−5^	1.69 × 10^−2^	1.56 × 10^−5^	6.48 × 10^−3^
**UXS1**	2	106,709,759	106,810,795	1.39 × 10^−4^	4.78 × 10^−2^	1.39 × 10^−4^	3.52 × 10^−2^
**ZNF197**	3	44,626,380	44,689,963	7.92 × 10^−5^	3.28 × 10^−2^	2.04 × 10^−5^	1.23 × 10^−2^
**KIAA1109**	4	123,073,488	123,283,913	1.51 × 10^−5^	9.73 × 10^−3^	2.06 × 10^−5^	1.44 × 10^−2^
ERAP2	5	96,211,643	96,255,420	5.49 × 10^−6^	2.16 × 10^−3^	1.03 × 10^−6^	8.68 × 10^−4^
LNPEP	5	96,271,098	96,373,219	3.53 × 10^−6^	2.36 × 10^−3^	1.50 × 10^−6^	9.68 × 10^−4^
**RGS14**	5	176,784,838	176,799,602	1.02 × 10^−6^	1.97 × 10^−3^	1.07 × 10^−5^	9.71 × 10^−3^
AHI1	6	135,604,670	135,818,914	1.98 × 10^−6^	2.61 × 10^−3^	3.31 × 10^−5^	1.10 × 10^−2^
**STEAP1B**	7	22,459,063	22,672,544	1.23 × 10^−5^	5.29 × 10^−3^	2.86 × 10^−5^	2.07 × 10^−2^
**PSMB7**	9	127,115,745	127,177,723	4.84 × 10^−5^	2.28 × 10^−2^	1.10 × 10^−4^	3.84 × 10^−2^
**TNFSF15**	9	117,546,915	117,568,406	4.03 × 10^−5^	1.60 × 10^−2^	4.09 × 10^−5^	1.61 × 10^−2^
ACTA2	10	90,694,831	90,751,147	5.64 × 10^−6^	4.05 × 10^−3^	1.03 × 10^−6^	7.17 × 10^−4^
FAS	10	90,750,414	90,775,542	5.21 × 10^−5^	2.32 × 10^−2^	1.08 × 10^−6^	8.93 × 10^−4^
**APOBR**	16	28,505,970	28,510,291	6.52 × 10^−7^	8.61 × 10^−4^	1.25 × 10^−6^	9.48 × 10^−4^
ATP2A1	16	28,889,726	28,915,830	5.31 × 10^−7^	1.18 × 10^−3^	3.66 × 10^−7^	5.87 × 10^−4^
**ATXN2L**	16	28,834,356	28,848,558	8.15 × 10^−6^	5.37 × 10^−3^	1.03 × 10^−6^	7.17 × 10^−4^
CLN3	16	28,477,983	28,506,896	5.82 × 10^−8^	2.53 × 10^−4^	2.43 × 10^−8^	3.38 × 10^−5^
**IL27**	16	28,510,683	28,523,372	2.10 × 10^−7^	4.10 × 10^−4^	3.93 × 10^−8^	1.30 × 10^−4^
**LAT**	16	28,996,147	29,002,104	1.53 × 10^−4^	5.40 × 10^−2^	4.62 × 10^−7^	6.13 × 10^−4^
**NFATC2IP**	16	28,962,128	28,978,418	1.93 × 10^−8^	9.20 × 10^−5^	8.07 × 10^−8^	1.84 × 10^−4^
**NUPR1**	16	28,548,606	28,550,495	6.39 × 10^−8^	3.04 × 10^−4^	1.94 × 10^−8^	4.69 × 10^−5^
**SBK1**	16	28,303,840	28,335,170	2.55 × 10^−7^	4.39 × 10^−4^	7.70 × 10^−7^	7.45 × 10^−4^
**SH2B1**	16	28,857,921	28,885,526	5.91 × 10^−9^	5.13 × 10^−5^	7.76 × 10^−7^	1.10 × 10^−3^
SULT1A1	16	28,616,903	28,634,946	1.54 × 10^−9^	8.17 × 10^−6^	8.27 × 10^−8^	1.25 × 10^−4^
SULT1A2	16	28,603,264	28,608,430	7.19 × 10^−11^	7.63 × 10^−7^	3.63 × 10^−8^	7.79 × 10^−5^
**TUFM**	16	28,853,732	28,857,729	2.12 × 10^−6^	2.05 × 10^−3^	4.10 × 10^−7^	7.41 × 10^−4^
**GSDMB**	17	38,060,848	38,076,107	6.69 × 10^−5^	2.64 × 10^−2^	2.22 × 10^−5^	1.16 × 10^−2^
**IKZF3**	17	37,921,198	38,020,441	4.01 × 10^−5^	1.69 × 10^−2^	2.70 × 10^−5^	9.35 × 10^−3^
**ORMDL3**	17	38,077,294	38,083,854	4.99 × 10^−5^	1.38 × 10^−2^	1.64 × 10^−5^	6.66 × 10^−3^
TYK2	19	10,461,209	10,491,352	5.12 × 10^−6^	4.05 × 10^−3^	1.94 × 10^−7^	2.01 × 10^−4^

22 novel gene were shown in bold. Gene position (start and end) are based on GRCh37/hg19 by Ensembl.

## Data Availability

The GWAS summary data analyzed during the current study are available in the NHGRI-EBI GWAS Catalog (https://www.ebi.ac.uk/gwas/downloads/summary-statistics, accessed on 9 November 2021) (Study Accession Code GCST90010715). The FUSION software and GTEx v8 gene expression datasets used during the current study are available in the FUSION website (http://gusevlab.org/projects/fusion/, accessed on 8 April 2022). The eMAGMA software and GTEx v8 annotion datasets used during the current study are available in the eMAGMA website (https://github.com/eskederks/eMAGMA-tutorial, accessed on 28 March 2022).

## Supplementary Materials

The following supporting information can be downloaded at: https://www.mdpi.com/article/10.3390/ijms232113555/s1.
